# Evaluation of Japanese people's perception of risk information for making decisions to receive influenza and rubella vaccinations

**DOI:** 10.1111/hex.13342

**Published:** 2021-08-25

**Authors:** Natsuko Yasuhara, Sawako Okamoto, Miki Hamada, Keita Uehara, Naoya Obana, Tomoaki Imamura

**Affiliations:** ^1^ Department of Public Health, Health Management and Policy Nara Medical University Nara Japan; ^2^ Social and Public Management Research Division Mitsubishi Research Institute, Inc. Tokyo Japan; ^3^ Present address: Natsuko Yasuhara, MSc, RN, Occupational Health Nurse, Resona Bank, Limited. Health Management Centre, Bingomachi 2‐2‐1, Chuo‐ku, Osaka‐shi, Osaka 540‐8610, Japan.Email: natsukology@gmail.com; ^4^ Present address: Keita Uehara, Manager, ClipLine, Inc., Customer Success, 22‐17, Nishigotanda 7‐Chome, Shinagawa‐Ku, Tokyo 141‐0031, Japan. Email: keita.uehara@clipline.jp

**Keywords:** barrier, influenza, intention, risk, rubella, vaccination

## Abstract

**Background:**

Generally, vaccination uptake in Japan lags behind World Health Organization targets.

**Objective:**

This study aimed to understand how risk information and advice affect intention to receive vaccinations.

**Methods:**

This study had a within‐subjects design. An online survey based on the Health Belief Model was sent to 2501 Japanese individuals (≧20 years) to assess the intention to be vaccinated for influenza and rubella after receiving minor and severe risk information and hypothetical advice about each vaccine. Regression analysis was used to measure changes in intentions to receive each vaccination after being provided with (1) risk information about each vaccine and (2) hypothetical encouragement and discouragement to be vaccinated.

**Main Outcomes:**

The main outcomes included changes in vaccination intentions from baseline.

**Results:**

Forty‐one percent (*N* = 1030) of those sent the survey completed it. At baseline, 43% and 65% of the respondents intended to have influenza and rubella vaccinations, respectively. Being provided with information about severe risks and susceptibility increased the intention to have the influenza vaccination among females in their 40s. Receiving inaccurate and discouraging information from one's mother significantly decreased the intention to have the rubella vaccination. Women 50 and older were more likely to intend not to have vaccination for rubella. Severe risk information decreased rubella vaccination intention in all age groups, except women in their 30s and 40s (*p* < .05).

**Conclusion:**

For both vaccinations, older individuals demonstrated vaccine hesitancy. This group requires tailored messaging to help them understand their vulnerability (to influenza) and their role in transmission (for rubella) to encourage uptake of essential vaccinations.

**Patient or Public Contribution:**

Members of the Japanese public responded to our online questionnaire on vaccination risk.

## 1 INTRODUCTION

In Japan, influenza vaccination rates among different age groups of adults older than 20 years ranged between 40% and 50% during the 2017/2018 influenza season; rubella vaccination coverage among different age groups ranged between 29% and 90% in 2018.^1^, ^2^ Both rubella and influenza vaccinations are recommended by the Japanese government, but vaccination decisions are voluntary. Although several previous reports describe factors that might increase Japanese people's vaccination intentions, they focused primarily on sociodemographic factors, understanding of vaccination benefits, physician recommendations and concerns about side effects.^3^, ^4^, ^5^ Forces that shape risk perceptions and methods that effectively promote vaccination uptake are not well understood in Japan.

Multiple studies have identified that concerns about the risks of receiving vaccinations, including side effects, pose a barrier to vaccination.^6^, ^7^, ^8^, ^9^, ^10^ Even if due to incorrect information, mistrust or misunderstanding, vaccine hesitancy dampens immunisation uptake.^9^, ^10^, ^11^ Documented primarily in the United States and Europe,^10^, ^12^ vaccine hesitancy appears to be multifactorial, attributable to underestimation of susceptibility, excessive anxiety about vaccine side effects and misinformation.^10^, ^11^, ^12^ Recent studies indicate that vaccine hesitancy is also operative in Japan,^11^ shaped by an anti‐vaccination movement that undermines confidence in vaccines and weakens government recommendations, a preference for treating diseases rather than preventing them,^13^ the costs of vaccination^3^ and insufficient information from healthcare services.^4^, ^5^

Risk perception is considered to be a combination of probability and subjective judgement shaped by an individual's unique psychological, social, cultural and even political context.^14^, ^15^ Matsui et al.^4^ found that accurate information and understanding of individual susceptibility, vaccination and severity of seasonal influenza infection were associated with increased vaccination uptake. However, even when respondents feel sufficiently informed to decide whether to be vaccinated, concerns about safety may shape risk perceptions and uptake, as demonstrated by a study by Walter et al.^16^ of a vaccination developed to combat the influenza A(H1N1)pdm09 pandemic. Social influences and access to services may also shape vaccination uptake.^4^, ^17^ However, how Japanese people perceive the risks of vaccination and how information and advice shape their intentions are still unknown.

For rubella, one would expect higher vaccination intentions among women of childbearing age. In Japan, rubella vaccination was mandatory for junior high students from 1977 to 1989, after which it became voluntary and vaccine coverage rates decreased, leading to rubella epidemics in the early 2000s. Since 2006, rubella vaccination guidelines recommend two childhood vaccinations: At 12 months of age and immediately before entering elementary school. The Ministry of Health, Labour and Welfare (MHLW) recommends rubella vaccination for adults, including healthcare workers who have not had a rubella vaccination in many years, those who are unsure whether they been previously infected with or vaccinated against rubella and those who plan to travel overseas.^18^ The Japanese government recommends that those who live with or have frequent contact with women of childbearing age be vaccinated for rubella because epidemics of rubella have occurred since 2002.^18^, ^19^

The aims of this study were to discover how exposure to different risk messages and social cues affect intentions to be vaccinated for influenza and rubella in Japan. Identification of differences in vaccination intentions between influenza and rubella could reveal how Japanese people appraise the risks of side effects alongside the benefits of being vaccinated. We designed risk messages around likely concerns about vaccination, including adverse reactions, consequences of remaining unvaccinated and social cues to action.^6^, ^8^, ^10^, ^14^, ^15^

## 2 METHODS

### 2.1 Research design

We designed an online survey administered by Macromill Inc. in Japan from 11 to 31 March 2014. Macromill Inc. is a private marketing research company that maintains a database of layperson registrants in Japan interested in participating in different research surveys. We selected Macromill Inc. to distribute the questionnaire and collect responses; their national reach of participants, experience with online survey research and their ability to build in cut‐off criteria suited our study design. The survey was designed to solicit intentions around receiving two elective vaccinations: seasonal influenza and rubella vaccinations. Both are voluntary in Japan; however, Japanese immunisation law states that influenza vaccines be generally obtained at individual expense, preferably annually and timed appropriately to provide immunity during the peak influenza season, while the rubella vaccination is strongly recommended and provided free, with resultant immunity persisting for many years.

To minimize bias in respondent risk perception about influenza and rubella, we avoided the peak influenza season. A within‐subjects design was used for the analyses; individuals under 20 years of age and medical professionals were excluded from the study. Variables included (1) demographics, (2) information‐seeking behaviours regarding healthcare‐ and vaccination‐related risks and (3) responses after receiving different risk information. Demographic questions included gender, age and education level to assess how these strata interpret the risks of vaccination. We also included having/not having a child or children under 20 years of age, based on evidence that having children affects influenza vaccination uptake.^3^

We developed risk statements around influenza and rubella vaccinations for the survey guided by the Health Belief Model (HBM).^20^ In the HBM, core constructs of perceived susceptibility to and severity of an adverse outcome, perceived benefits of and barriers to adopting a preventive action, cues to action and self‐efficacy all shape intentions. If perceived benefits of a preventive action exceed perceived barriers, and if individuals perceive that they are susceptible to an adverse outcome, they are more likely to adopt a recommended preventive health action. In this study, we defined the adverse outcome as side effects of having vaccination, because several articles reported that concerns about side effects of having vaccination would lead to a lack of confidence or negative attitude towards vaccination.^6^, ^8^, ^9^, ^10^ We defined ‘severity’ as the severity of the side effect, ‘susceptibility’ as the risk of experiencing infections and ‘barrier’ to adopt a preventive action as a barrier to having vaccination. If respondents saw vaccination as beneficial after being made aware of minor and severe risks and susceptibility, their intention to receive a vaccination would subsequently increase. Cues to action, such as messages in mass media campaigns, doctors' recommendations and advice from others, can also promote or discourage vaccination intentions. Self‐efficacy is the level of a person's confidence in his or her ability to take action^21^ and is associated with increased information‐seeking behaviours as well as integration and more effective use of information.^22^

### 2.2 Survey design

First, respondents were asked about their intentions to receive influenza and rubella vaccinations, when provided no information about either vaccination. Then, respondents were asked about their intentions to receive each vaccination after being provided different levels and types of information: Description of the risk of a minor adverse reaction, description of the risk of a severe adverse reaction and an adverse health risk faced by unvaccinated persons (susceptibility). Self‐efficacy was assessed through questions inquiring about other health information‐seeking behaviours. For cues to action, we asked respondents to consider two hypothetical nonmedical advice statements: (1) being discouraged by one's mother from getting the vaccine and (2) being encouraged to get the vaccine by a friend. These scenarios were selected based on studies showing that parental advice (especially from one's mother) and peer influences have a strong influence on preventive health behaviours and vaccination uptake among young adults of childbearing age.^23^, ^24^, ^25^, ^26^ Peers have been shown to influence decision‐making by parents about immunisations for their children.^24^, ^26^ It is unclear whether and how maternal influence over health decision‐making wanes over the life course in Japan; our study attempted to address this literature gap by exploring whether and how maternal and peer advice influenced adult intentions.

Minor and severe risk explanations were designed by the authors based on information published on the home page of the MHLW, including the National Institute of Infectious Diseases (Table 1). The information on the MHLW website is based on medical evidence, but worded for laypersons, with some probabilities presented as percentages, while others are qualitative, using phrases such as ‘in quite rare cases’. Conveying vaccination risk information in this way seems to be common practice internationally, exemplified by information issued by the Centres for Disease Control and Prevention (CDC) in the United States.

**Table 1:** Risk information in the online survey

	Influenza vaccination	Rubella vaccination
Minor risk	10%–20% of people receiving the influenza vaccination will experience eczema at the injection site. 5%–10% will have eczema all over the body, but it will resolve in 2–3 days	One in several thousand people (0.05%) receiving the rubella vaccination will experience severe headache with cramping at the back of the neck, fever, nausea and/or vomiting (aseptic meningitis). For comparison, the all‐cause rate of aseptic meningitis among those not vaccinated for rubella is 2 in 100 (2%)
Severe risk	In rare cases, fever, headache, spasms, disturbance of motility and/or consciousness, shock, hives and difficulty breathing may occur several days to 2 weeks after having the influenza vaccination. These side effects may be serious and possibly life‐threatening	In rare cases, idiopathic thrombocytopenic purpura may develop, which poses a risk of mild to excessive bruising and bleeding. This condition is associated with an unusually low level of platelets, which aid blood clot. Extremely rarely, platelet levels may fall so low that dangerous internal bleeding occurs, though effective treatments are available
Susceptibility	Very rarely, unvaccinated individuals may develop bronchitis, pneumonia and/or encephalitis. Cardiac arrest may occur; the risk is higher among seniors and those with chronic conditions	If pregnant women who are not immune to rubella are infected, their babies may be born with birth abnormalities, such as hearing difficulties, cataracts and cardiac deformity (congenital rubella syndrome). The frequency of birth abnormalities caused by rubella infection during pregnancy is 1.8–7.7 per 100,000 births (0.002%–0.008%). The frequency of birth abnormalities by weeks of pregnancy when the infection occurs is more than 50% at 4 weeks' gestation and about 35% at 8 weeks' gestation
Cue to action (mother's discouraging comment)	Your mother said that you do not need to have the vaccination because even if you contract it, you will recover by just staying in bed for several days	Your mother said that you do not need to have the vaccination because she believes that you already contracted rubella in childhood
Cue to action (friend's encouraging comment)	One of your friends said that they would have the vaccination if it would prevent or alleviate the symptoms of rubella	One of your friends said that you should get vaccinated because your belief that you had rubella as a child might be mistaken

We piloted the survey to measure the reliability of the questionnaire using a test–retest approach, adapting the questions to reach a sufficiently high Cronbach's *α* (*α* = .90) with a subgroup of respondents: .89 for the influenza vaccination and .82 for the rubella vaccination.

At baseline and after being presented with each explanation of risk or cue to action, respondents rated their intentions to receive each vaccine along a 6‐point Likert scale, where 1 indicated no intention of having a vaccination and 6 indicated strong intention to have a vaccination. The results were dichotomized so that scores of 1, 2 and 3 were converted into 0 (*no intention*) and scores of 4, 5 and 6 were converted into 1 (*intention*).

### 2.3 Data analysis

Respondents' vaccination intentions were examined using multiple logistic regression analyses using stated intentions before being provided any information as a baseline to compare with intentions after being provided different risk information and advice. McNemar tests were used to compare baseline intentions with intentions after being provided risk and nonmedical information. The level of significance was set at 5%. IBM SPSS Statistics version 25.0 was used for all statistical analyses.

### 2.4 Ethical considerations

Participation in this study was voluntary and anonymous; individuals could not be identified by researchers. Respondents were asked if they would like to participate in the online survey, and the questionnaire was distributed only to those who agreed for it to be sent. Agreeing to receive a questionnaire did not constitute consent to participate. Respondents who entered the survey site were considered to have given agreement and informed consent after they clicked to agree to participate and submitted their answers; they were free to opt out at any time. This study was approved by our institution's Ethics Committee (Approval #821).

## 3 RESULTS

### 3.1 Participant profile

The questionnaire was electronically distributed to 2501 individuals. Of these, 1030 respondents (41%) completed the survey, of whom 515 were male and 515 were female. Macromill implemented an automatic cut‐off for enrolment in each age group to ensure a 1:1 ratio of males to females and an even distribution among the five age groups: 20s, 30s, 40s, 50s and 60s and older (*n* = 103 per group). The respondents' mean age was 45 years (*SD*: ±14.65). Slightly more than approximately half (*n* = 556, 54%) had attended a 2‐year college or higher. Approximately one‐third of the respondents had one or more children under 20 years of age (*n* = 297, 29%). At baseline, before being provided any risk information, 440 (43%) stated an intention to have the influenza vaccination and 665 (65%) stated an intention to have the rubella vaccination.

Exploratory factor analysis was performed on three groups of factors: (1) information‐seeking behaviours, (2) preinformed vaccination intentions (baseline) and postinformed vaccination intentions (minor risk, severe risk, susceptibility, mother's discouragement and friend's discouragement) for influenza vaccinations and (3) pre‐ and postinformed vaccination intentions for rubella vaccinations.

### 3.2 Comparison of vaccination uptake intentions

#### 3.2.1 Influenza vaccination

##### 3.2.1.1 Intentions to have influenza vaccination (multiple logistic regression analyses)

No gender association was found with intention to have the influenza vaccination. Overall, respondents in their 40s and 50s, regardless of gender, did not intend to have the influenza vaccination at baseline; intentions did not change after risk information or advice was presented (*p* < .05 for all associations; Table 2; Table S1).

**Table 2:** Intention to have influenza and rubella vaccinations (multiple logistic regression analysis: *n* = 1030)

		No information (baseline)		Minor risk		Severe risk		Susceptibility		Maternal discouragement		Friend's encouragement	
		Adjusted odds ratio (95% CI)	*p* Value	Adjusted odds ratio (95% CI)	*p* Value	Adjusted odds ratio (95% CI)	*p* Value	Adjusted odds ratio (95% CI)	*p* Value	Adjusted odds ratio (95% CI)	*p* Value	Adjusted odds ratio (95% CI)	*p* Value
Influenza vaccination intention													
Age													
20–29 (Ref.)			<.01		<.01		<.01		<.01		<.01		<.01
30–39		1.21 (0.82, 1.81)	.34	1.02 (0.68, 1.51)	.94	1.19 (0.80, 1.77)	.39	1.15 (0.77, 1.71)	.50	1.18 (0.80, 1.76)	.41	0.99 (0.67, 1.48)	.97
40–49		0.73 (0.49, 1.11)	.14	**0.56 (0.37, 0.84)** **	<.01	**0.62 (0.41, 0.94)** *	.02	**0.52 (0.35, 0.79)** **	<.01	0.63 (0.42, 0.95)*	**.03**	**0.58 (0.39, 0.87)** **	.01
50–59		**0.61 (0.41, 0.91)** *	.02	**0.49 (0.33, 0.73)** **	<.01	**0.55 (0.37, 0.82)** **	<.01	**0.48 (0.32, 0.71)** **	<.01	**0.55 (0.37, 0.83)** **	<.01	**0.55 (0.37, 0.82)** **	<.01
60 and over		1.22 (0.83, 1.81)	.32	0.87 (0.59, 1.28)	.47	0.94 (0.63, 1.39)	.75	0.83 (0.56, 1.23)	.35	0.97 (0.66, 1.44)	.89	1.00 (0.68, 1.48)	.99
Gender													
Male (Ref.)													
Female		0.88 (0.69, 1.14)	.34	0.87 (0.68, 1.12)	.28	0.83 (0.64, 1.06)	.14	0.88 (0.69, 1.13)	.32	0.88 (0.68, 1.13)	.31	0.93 (0.73, 1.20)	.58
Has child/children < 20 years													
No (Ref.)													
Yes		1.37 (1.91, 1.85)	.05	1.29 (0.95, 1.74)	.11	1.47 (1.08, 1.99)*	.01	**1.38 (1.02, 1.88)** *	.04	**1.42 (1.05, 1.92)** *	.03	**1.52 (1.12, 2.06)** **	.01
Education level													
High school or less (Ref.)													
More than high school		1.19 (0.92, 1.53)	.18	1.22 (0.95, 1.57)	.12	1.24 (0.96, 1.59)	.10	1.13 (0.88, 1.45)	.34	1.12 (0.87, 1.44)	.38	1.11 (0.86, 1.43)	.41
Rubella vaccination intention													
Age													
20–29 (Ref.)			<.01		**.01**		.02		<.00		<.01		<.01
30–39		0.65 (0.42, 1.01)	.58	0.71 (0.46, 1.10)	.13	0.70 (0.46, 1.07)	.10	**0.60 (0.39, 0.94)** *	.03	0.67 (0.44, 1.01)	.06	0.75 (0.50, 1.12)	.16
40–49		**0.44 (0.29, 0.69)** **	<.01	**0.52 (0.33, 0.80)** **	<.01	**0.54 (0.35, 0.82)** **	<.01	**0.47 (0.30, 0.74)** **	<.01	**0.47 (0.31, 0.74)** **	<.01	**0.43 (0.29, 0.66)** **	<.01
50–59		**0.40 (0.26, 0.62)** **	<.01	**0.51 (0.34, 0.78)** **	<.01	0.64 (0.43, 0.96)*	**.03**	**0.52 (0.34, 0.79)** **	<.01	**0.45 (0.30, 0.70)** **	<.01	**0.54 (0.36, 0.79)** **	<.01
60 and over		**0.49 (0.32, 0.75)** **	<.01	**0.52 (0.34, 0.79)** **	<.01	**0.54 (0.36, 0.81)** **	<.01	**0.48 (0.32, 0.74)** **	<.01	**0.47 (0.31, 0.72)** **	<.01	**0.52 (0.35, 0.78)** **	<.01
Gender													
Male (Ref.)													
Female		1.05 (0.81, 1.37)	.69	0.89 (0.69, 1.16)	.40	1.00 (0.77, 1.29)	.98	1.22 (0.94, 1.59)	.14	**0.66 (0.50, 0.87)** **	<.01	0.79 (0.61, 1.02)	.07
Has child/children < 20 years													
No (Ref.)													
Yes		1.20 (0.87, 1.65)	.27	1.40 (1.01, 1.93)*	.04	**1.59 (1.16, 2.18)** **	<.01	**1.89 (1.35, 2.64)** **	<.01	1.05 (0.75, 1.47)	.77	1.01 (0.81, 1.49)	.55
Education level													
High school or less (Ref.)													
More than high school		1.27 (0.98, 1.64)	.75	1.17 (0.90, 1.52)	.24	1.17 (0.91, 1.51)	.23	**1.34 (1.03, 1.75)** *	.03	1.09 (0.82, 1.44)	.56	1.13 (0.87, 1.45)	.37

*Note:* gender: male = 0, female = 1; age: 20–60s and over; having a child/children: yes = 1, no = 0; education level: more than high school = 1, high school or less = 0. Bold values indicate statistically significant values.

Abbreviation: CI, confidence interval.

* *p* < .05.

** *p* ≤ .01.

As for statistical interactions, females in their 40s had higher influenza vaccination intentions after receiving severe risk information (*p* = .02, 34 of 103, 33%) and susceptibility information (*p* = .01, 37 of 103, 36%). Conversely, females 60 and older showed no intention of having influenza vaccination at baseline (*p* = .02, 57 of 103, 55%); intentions remained unchanged by provision of any risk information (*p* < .05 for all associations). Those in their 40s who had children intended to have the influenza vaccination at baseline (48 of 111, 43%) and after any risk information was provided (*p* < .05 for all associations). Those in their 30s with children indicated an intention to be vaccinated only when provided severe risk information (*p* = .04, 63 of 97, 65%). Highly educated respondents in their 30s and 40s intended to have the vaccination at baseline (64 of 118 [54%] and 44 of 102 [43%], respectively) and after any risk information was provided (*p* < .05 for all associations; Table S2).

##### 3.2.1.2 Comparison of intentions between baseline and after provision of different risk information and advice (McNemar tests)

Intentions to have the influenza vaccination significantly increased from baseline after being provided minor risk information (9% increase, *n* = 41 more than baseline), susceptibility information (10% increase, *n* = 42 more than baseline) and a friend's comment (8% increase, *n* = 34 more than baseline; *p* < .01 for all associations). Even severe risk information and a mother's discouragement did not influence the respondents' vaccination intentions (Table S3).

#### 3.2.2 Rubella vaccination

##### 3.2.2.1 Intentions to have rubella vaccination (multiple logistic regression analyses)

Overall, gender was not associated with intention to receive the rubella vaccination. However, females (*n* = 515) were significantly more likely to refuse the rubella vaccination after being hypothetically discouraged by their mother (*p* < .01, 76%, *n* = 390; Table S1). Regarding statistical interactions, females in their 30s (*n* = 103) and 40s (*n* = 103) were significantly more likely than other age groups to intend to have the vaccination at baseline (74%, *n* = 77 and 59%, *n* = 61, respectively) and after any risk information was provided (*p* < .05 for all associations). However, females 50 and older (*n* = 206) were statistically significantly more likely than other groups to refuse the rubella vaccination after a mother's discouragement (83%, *n* = 172; both, *p* < .05).

Both education and stage of life had a huge impact on intention to be vaccinated, even at baseline. Women with children under 20 years (*n* = 168), respondents in their 40s with children (*n* = 111), highly educated respondents with children (*n* = 158) and highly educated respondents in their 40s (*n* = 102) all responded to information about minor risk with slight increases in intention to have the vaccine (1%, 7%, 3% and 3%, respectively). Severe risk information decreased intention somewhat for most of these groups (2%–3% decrease among women with children, highly educated respondents with children and highly educated respondents in their 40s), though there was a 3% increase in intention for respondents in their 40s with children (*p* < .05 for all associations). Susceptibility information seemed to generate larger increases in intention than any risk information about having the vaccination (7% increase among women with children, 9% increase among those in their 40s with children, 4% increase among highly educated respondents with children and 6% increase among highly educated respondents in their 40s; *p* < .05 for all associations; Table S4).

##### 3.2.2.2 Comparison of intentions between baseline and after provision of different risk information and advice (McNemar tests)

A number of inputs significantly decreased intentions to have the rubella vaccination from baseline, including severe risk information (7% decrease, *n* = 45 fewer than baseline), hypothetical maternal discouragement (56% decrease, *n* = 372 fewer) and even a friend's hypothetical encouragement to have the rubella vaccination (36% decrease, *n* = 242 fewer; *p* < .01 for all associations). Hearing a mother's discouragement first seemed to influence subsequent responses to a friend's encouragement. Therefore, a McNemar test was conducted on the results to assess the relationship between the mother's discouragement and the friend's encouragement. There was a significant increase in vaccination intention when the friend's encouragement was provided (44% increase, *n* = 130 more than after mother's discouragement; *p* < .01; Table S3).

#### 3.2.3 Comparison of information‐seeking behaviours with vaccination intentions

For our measure of self‐efficacy, approximately half of the respondents indicated that they engage in each of the health information‐seeking behaviours surveyed. Over half of the respondents (*n* = 577, 56%) stated that they would compare the advantages and disadvantages of a medical procedure if treatment were required; 49% (*n* = 509) reported reading the warning labels on over‐the‐counter flu medication, 49% (*n* = 508) stated that they researched the risks of having an influenza vaccination and 62% (*n* = 643) stated that they researched the risks of having a rubella vaccination. Elderly individuals, as well as females more generally, were statistically significantly more likely to seek vaccination information and assess the risks and benefits (Table S5).

##### 3.2.3.1 Influenza vaccination

Researching the risks of medical procedures was significantly related to intentions of having the influenza vaccination at baseline (27%, *n* = 275) and after being provided any risk information or comments (29%, *n* = 301 minor; 27%, *n* = 278 severe; 29%, *n* = 299 susceptible; 27%, *n* = 280 mother's comment; and 28%, *n* = 291 friend's comment; *p* < .01 for all associations). Those who researched the risks of the influenza vaccination had significantly higher influenza vaccination intentions after being provided any risk information (*p* = .02, 26%, *n* = 264 minor; *p* = .01, 24%, *n* = 244 severe; *p* = .04, 25%, *n* = 260 susceptible; Table S6).

##### 3.2.3.2 Rubella vaccination

Comparing the advantages and disadvantages of having medical procedures was significantly related to intentions of having the rubella vaccination at baseline (38%, *n* = 394), after being provided minor (39%, *n* = 399) and severe risk information (36%, *n* = 370) and a mother's discouragement (18%, *n* = 189; *p* < .05 for all associations). Those who had researched the risks of the rubella vaccination had significantly higher vaccination intentions even after being provided a mother's discouragement (*p* = .02, 21%, *n* = 213; Table S6).

## 4 DISCUSSION

### 4.1 Demographic aspects of vaccine hesitancy

The influenza vaccination is required annually, at personal expense and is self‐protective, particularly for the very young and elderly, while the rubella vaccination is needed much less frequently and offers direct, long‐lasting benefits primarily to women of childbearing age and children. Despite these differences, older respondents expressed more vaccine hesitancy towards both vaccinations than younger respondents. Among older respondents, intentions to have either vaccination were low at baseline and were largely unaffected by provision of any risk or susceptibility information or opinions of their peers. Japanese seniors generally seemed to have already made up their minds not to have the influenza vaccination, fitting the complacency model of vaccine hesitancy.^10^, ^11^, ^12^ Similarly, older individuals were generally uninterested in rubella vaccination, seemingly oblivious to the risks of being infected and spreading rubella to others who are not vaccinated. This resistant attitude poses a considerable barrier to efforts by the Japanese government to increase uptake and is of public health concern, as the elderly are at a much higher risk of serious illness and death from influenza if unvaccinated, and can spread rubella infection.^13^, ^18^ Surprisingly, this demographic group also reported being proactive in obtaining vaccination information and considering its benefits, but despite this apparent self‐efficacy, they were still impervious to vaccination uptake.

Those in their 30s and 40s—particularly females, those with children and highly educated respondents—were more likely to express influenza vaccination intentions after receiving information on risk (even severe risk) and susceptibility. It is possible that this demographic, most likely to have young children at home, has better information about vaccination benefits and more favourable attitudes to vaccination based on their children's vaccination experiences at their paediatrician and/or at school. Additionally, connections made with other parents through their children would allow for an exchange of information, possibly leading to an increase in vaccination uptake.

### 4.2 Peer and family influences

Remarkably, even for older generations whose mothers are likely infirm or deceased, maternal influence was stronger than any of the medical information provided about the rubella vaccination. Japanese culture and family patterns may influence these responses. In East Asian cultures including Japan, people tend to respect the opinions of their elders because of their life experience^27^ and also view themselves as interdependent with others in specific contexts.^28^ Respecting one's elders still influences older generations: knowing that one's mother had a negative opinion of vaccination could decrease vaccination intention. However, considering that we did not observe any maternal influence on influenza intention among older age groups, maternal discouragement might instead provide a convenient excuse for refusal of the rubella vaccination, which would lead to vaccine hesitancy due to complacency.^10^, ^11^, ^12^

Hypothetical advice from one's mother was intentionally based on a potentially unreliable source (her memory) as well as medically incorrect information. Regardless, a sizeable proportion of respondents of all ages reported that this advice would decrease their intention to receive the rubella vaccination. Respondents who were influenced by maternal advice appeared to accept vaccination information unquestioningly. A previous study has reported that parental influence, especially maternal influence, affects young adults' decision‐making about preventive vaccinations for women's diseases.^25^ Focusing on increasing support for rubella vaccination among mothers, especially mothers of grown children, may boost vaccination intention in those of reproductive age, but further research is required.

Peer influence on younger generations was not particularly evident in this study, as peer encouragement did not seem to increase vaccination intention in younger cohorts as it did among the middle‐aged with children at home, who are more likely to be exposed to information and opinions through school activities through their children and parental meetings at school. Peer influence seemed to have no effect on rubella vaccination intentions. Those with high information‐seeking behaviours generally had higher vaccination intentions, especially for influenza. An exception was the elderly, who had high health information‐seeking behaviour, but lower vaccination uptake. High self‐efficacy among the elderly appeared to be influenced or reinforced by peers; therefore, providing information and vaccinations in gathering places like existing community resources for seniors might enhance vaccine uptake.^29^

### 4.3 Effect of informational inputs and sequencing on vaccine intentions

We examined intentions around vaccination for two very different vaccinations. Barriers to action are logically much higher to achieve personal and population protection when it comes to influenza vaccines than rubella, given the out‐of‐pocket cost and frequency of vaccination required. Providing information based on increasing risk level helps people better appraise differences in risk. The MHLW home pages describing the influenza and rubella vaccinations describe their risks in the side effects section, not in conjunction with information about susceptibility to disease or the benefits of vaccination. In our study, information on both the risks of side effects as well as susceptibility to adverse outcomes from getting the disease was provided sequentially, following the HBM categories, so that individuals could consider the benefits.

Providing risk information in a sequential manner seemed to effectively allow younger generations to rationally consider the advantages and risks of having vaccinations, especially for influenza. In contrast, older generations indicated low intention to have either vaccination at baseline, and presentation of medical information about vaccination risks and their susceptibility did not change their minds, despite high self‐efficacy around health decisions. Vaccine hesitancy in the elderly should be a focus of future research.

### 4.4 Implications for vaccination campaigns in Japan

In Japan, a high‐income nation with a well‐developed vaccination programme, vaccine hesitancy may be driven by mistaken impressions about vaccinations, some of which may be driven by misinformation provided by peers or on the Internet; however, poor messaging, changing recommendations and outreach by vaccination campaigns may also play a role.^3^, ^4^, ^5^, ^13^ The Japanese Health Department (MHWL) seemed to have provided important information about vaccines only sporadically.

For both vaccinations, those aged 50 and older showed high vaccine hesitancy and were unswayed by information in our survey, despite self‐reported high levels of health information‐seeking behaviours and widespread availability and accessibility of vaccinations. High vaccine hesitancy in this vulnerable group is problematic, and further research will be needed to develop appropriate messages to motivate older vaccination recipients. Other factors may also encourage vaccine hesitancy in older age groups, who are more likely than others to encounter difficulty due to cognitive and physical mobility issues in accessing and paying for vaccines. Our study design did not tailor risk information by age group, but highlighting elderly individuals' higher susceptibility to adverse outcomes of influenza infection in vaccination messages may be one avenue to increase influenza vaccination rates in Japan. Involving family physicians in vaccine campaigns might complement information provided by national campaigns, help the elderly better understand the need for each vaccination and address any concerns about the side effects of vaccination. Higuchi et al.^5^ showed that family physician recommendation was positively correlated with pneumococcal vaccination intention among the elderly in a rural area of Japan.

The Japanese government has promoted rubella vaccination primarily by focusing on the risk of congenital rubella syndrome in unborn children—an important benefit to women of childbearing age and their children. However, this emphasis may have inadvertently contributed towards indifference among those older than reproductive age. Sensitizing older individuals to understand that if they are infected, they could spread rubella to pregnant unvaccinated females and cause birth defects in children might motivate them to get vaccinated. Overcoming rubella vaccine hesitancy among these populations in Japan may require messages that help this group view vaccination uptake as a prosocial contribution.

### 4.5 Limitations

Use of an Internet survey excluded individuals who do not have online access or do not use computers or smartphones, potentially under‐representing the very poor and very old. The survey relied on self‐report, which may be unreliable and could include some social desirability bias (e.g., respondents may have over‐reported intention to be vaccinated before receiving any vaccine information, which would diminish the true effect of providing risk information). Using a private marketing firm's database for the study population relied on their roster of registered participants, which may introduce some selection bias. However, the vast majority of Japanese people are highly computer literate, and the large number of responses across Japan indicates that geographic reach was good.

Other limitations pertain to study design and analysis. Due to changing policies and recommendations around vaccination, the proportions of unvaccinated individuals are not evenly distributed by age group in Japan, particularly for rubella, and some respondents probably had had adult rubella vaccination, which may have decreased the observed effect of the risk information provided. The way in which different types of information were provided (minor and severe risks, susceptibility, mother's and friend's advice) may have created an order or cumulative effect. Additionally, our questionnaire design made it impossible to ascertain whether the impact of advice from friends and family was attributable to the content of the advice or the relationship with the person giving the advice. However, within‐subjects designs have two advantages: Higher statistical power and a lower probability of failing to detect a true difference.

Subjective expressions such as ‘the severity is high’ and ‘the probability of the outcome is so low’ may have been understood differently by different respondents according to their risk tolerances and subjective interpretations of these phrases. Using congenital rubella syndrome as the example of susceptibility for the rubella vaccination may have skewed favourable intentions towards women of reproductive age. However, these are common ways to communicate vaccination risk information to the public used by major national healthcare organisations, such as the CDC and MHLW. Additional research is needed to evaluate how the citizens of Japan and other countries perceive these messages.

Our analytical decision to collapse our 6‐point Likert scales to a dichotomous scale introduced potential bias, but is a common approach in social research to allow for analysis of a discrete outcome of interest (in this case, vaccination intention or not). As Japanese people, like people from other East Asian nations, tend to choose the middle choices in questionnaires,^30^ dichotomization was an attempt to allow respondents to feel comfortable with their response while capturing meaningful responses.

## 5 CONCLUSION

Messages and information to increase vaccine uptake in Japan and elsewhere must target specific demographics with tailored approaches that target the main factors underlying vaccine hesitancy for specific vaccines and for each sociodemographic group, particularly the elderly, who show higher vaccine hesitancy than other groups despite higher susceptibility to many vaccine‐preventable diseases. Risk information about severe side effects tends to decrease vaccine intention and should be delivered carefully and in the proper context (e.g., alongside benefits and susceptibility) to groups most concerned about these risks. Messages to motivate individuals to receive vaccines that may not benefit them directly, such as for rubella vaccine promotion among men and older individuals, will require inventive approaches, possibly by appealing to the sense of shared social obligation that animates Japanese culture.

The findings of our study confirm studies from other East Asian countries that have identified high vaccine hesitancy, particularly among the elderly, for a range of different vaccinations. Our findings highlight the need for vaccine campaign strategies that identify segments of the populations where vaccine hesitancy is high and to develop culturally appropriate and effective messages and approaches tailored to the concerns and norms of different age and educational groups.

## CONFLICT OF INTERESTS

The authors declare that there are no conflict of interests.

## Supporting information

Supporting information.

## Data Availability

The data that support the findings of this study are available from the corresponding author upon reasonable request.
